# Dislocated hinge fractures are associated with malunion after lateral closing wedge distal femoral osteotomy

**DOI:** 10.1007/s00167-021-06466-2

**Published:** 2021-02-27

**Authors:** Marco-Christopher Rupp, Philipp W. Winkler, Patricia M. Lutz, Markus Irger, Philipp Forkel, Andreas B. Imhoff, Matthias J. Feucht

**Affiliations:** 1 Department of Orthopedic Sports Medicine, Klinikum Rechts der Isar, Technical University of Munich, Ismaninger Str. 22, 81675 Munich, Germany; 2 Department of Orthopaedics and Trauma Surgery, Medical Center, Faculty of Medicine, Albert-Ludwigs-University of Freiburg, Freiburg, Germany

**Keywords:** Closing wedge, Distal femoral osteotomy, Hinge fracture, Complications, Malunion, Classification, Varus deformity

## Abstract

**Purpose:**

To evaluate the incidence, morphology, and associated complications of medial cortical hinge fractures after lateral closing wedge distal femoral osteotomy (LCW-DFO) for varus malalignment and to identify constitutional and technical factors predisposing for hinge fracture and consecutive complications.

**Methods:**

Seventy-nine consecutive patients with a mean age of 47 ± 12 years who underwent LCW-DFO for symptomatic varus malalignment at the authors’ institution between 01/2007 and 03/2018 with a minimum of 2-year postoperative time interval were enrolled in this retrospective observational study. Demographic and surgical data were collected. Measurements evaluating the osteotomy cut (length, wedge height, hinge angle) and the location of the hinge (craniocaudal and mediolateral orientation, relation to the adductor tubercle) were conducted on postoperative anterior–posterior knee radiographs and the incidence and morphology of medial cortical hinge fractures was assessed. A risk factor analysis of constitutional and technical factors predisposing for the incidence of a medial cortical hinge fracture and consecutive complications was conducted.

**Results:**

The incidence of medial cortical hinge fractures was 48%. The most frequent morphological type was an extension fracture type (68%), followed by a proximal (21%) and distal fracture type (11%). An increased length of the osteotomy in mm (53.1 ± 10.9 vs. 57.7 ± 9.6; *p* = 0.049), an increased height of the excised wedge in mm (6.5 ± 1.9 vs. 7.9 ± 3; *p* = 0.040) as well as a hinge location in the medial sector of an established sector grid (*p* = 0.049) were shown to significantly predispose for the incidence of a medial cortical hinge fracture. The incidence of malunion after hinge fracture (14%) was significantly increased after mediolateral dislocation of the medial cortical bone > 2 mm (*p* < 0.05).

**Conclusion:**

Medial cortical hinge fractures after LCW-DFO are a common finding. An increased risk of sustaining a hinge fracture has to be expected with increasing osteotomy wedge height and a hinge position close to the medial cortex. Furthermore, dislocation of a medial hinge fracture > 2 mm was associated with malunion and should, therefore, be avoided.

**Level of evidence:**

Prognostic study; Level IV.

## Introduction

Varus malalignment has been demonstrated to play an important role in development of medial knee joint cartilage degeneration and thus predispose for medial knee osteoarthritis [11, 18, 29, 45, 52]. Hence, surgical correction of varus malalignment by valgus producing osteotomy to unload the medial compartment is indicated in moderate medial knee osteoarthritis [5, 18] or in combination with cartilage regenerative [1, 19] or meniscus replacing procedures [26, 27]. While historically, correction of varus alignment has been predominantly described via high tibial osteotomy (HTO) [5, 7, 16, 18, 22–24, 28, 30, 33, 34, 40, 49], the dogma of an isolated tibial based deformity is recently being reevaluated [13, 37]. To prevent joint line obliquity that negatively affects joint biomechanics [2, 37, 42, 46, 48, 51], it was shown that in 71% of the cases, a (concomitant) femoral correction is required [13]. While the favourable postoperative outcomes reported in the literature have increased the popularity of the femoral lateral closing (CW) wedge technique [2, 15, 25, 35, 43, 44], recent investigations are aimed to identify reasons for failures [13]. Hinge fractures in distal femoral osteotomies (DFO)—resulting in increased rotational movement across the osteotomy plane [3, 41]—may explain a considerable number of delayed unions or losses of correction reported [4, 6, 8, 9, 14, 25, 54]. While technical factors and vulnerable anatomical zones associated with an increased risk of hinge fractures have been reported for HTO [16, 31–33, 49], medial closing wedge (MCW) DFO [21, 39] and LOW-DFO [53], the only report investigating hinge fractures in LCW-DFO to-date was performed in a relatively small collective and could not identify any risk factors [36].

Thus, the primary objective of this retrospective observational study was to evaluate the incidence, morphology, and associated complications of medial cortical hinge fractures after LCW-DFO. The secondary objective was to identify demographic, constitutional or technical factors predisposing for a medial cortical hinge fracture and consecutive complications. It was hypothesised that medial cortical fracture in LCW-DFO is common, results in complications, and that a medial hinge position and a high degree of correction would increase the risk of sustaining a medial cortical hinge fracture.

## Methods

This was an Institutional-Review-Board (Technical University of Munich, No. 6/20S) -approved retrospective observational study. Ninety consecutive patients who underwent LCW-DFO for symptomatic varus malalignment between 01/2007 and 03/2018 were screened for eligibility. Preoperative anterior–posterior (AP) hip-knee-ankle radiographs and standard lateral radiographs as well as standard postoperative AP and lateral knee radiographs, comprehensive medical records and a minimum of 2 years of postoperative time interval were required for inclusion. Previous osteotomies, posttraumatic deformities of the distal femur, (concomitant) axis correction in the sagittal plane as well as a malrotation of the postoperative AP knee radiograph (resulting in a misprojection of the bony landmarks) were defined as exclusion criteria. Eleven patients meeting the exclusion criteria (2 severely malrotated radiographs, 1 posttraumatic deformity, 8 concomitant correction in the sagittal plane) were excluded. A total of 79 patients (mean age: 47 ± 12 years, male sex: 66%) were included in the final analysis. A LCW-DFO was performed for medial compartment osteoarthritis in 67 (85%), an osteochondral defect in the medial compartment in 9 (11%), and chronic ligamentous insufficiency in 3 (4%) cases as the respective main diagnosis. Detailed characteristics of the patient collective are presented in Table 1.

**Table 1:** Continuous variables are presented as mean ± standard deviation (range); Categorical variables are presented as count and percentage

Variable		Total study group
Number of included patients, *n*		79
Age^a^ (years)		47 ± 12 (16–80)
BMI (kg/m^2^)		28 ± 5 (45–18)
Sex		
Female, *n* (%)		27 (34%)
Male, *n* (%)		52 (66%)
Laterality		
Left, *n* (%)		46 (58%)
Right, *n* (%)		33 (42%)
Hinge fracture		
Yes, *n* (%)		38 (48%)
No, *n* (%)		41 (52%)
Fracture morphology^b^		
Type 1 (extension), *n* (%)		26 (68%)
Type 2 (distal), *n* (%)		4 (11%)
Type 3 (proximal), *n* (%)		8 (21%)
Concomitant procedures^c^		
None, *n* (%)		39 (49%)
HTO-MOW, *n* (%)		23 (29%)
Ligament surgery, n (%)		2 (3%)
OATS, *n* (%)		2 (3%)
Meniscus surgery, *n* (%)		15 (20%)
Cartilage transplantation, *n* (%)		2 (3%)

BMI, body-mass-index; HTO-MOW, high tibial osteotomy medial open wedge; OATS, osteochondral autograft transplant

^a^Age at surgery

^b^Hinge fracture group (*n* = 38)

^c^Total number of patients exceeds 79 (total study group), as certain patients underwent more than one concomitant procedure

### Indications and surgical technique

Varus malalignment was analysed on AP hip–knee–ankle radiographs prior to surgery and the osteotomy was planned employing mediCAD^®^ software (mediCAD Hectec GmbH, Altdorf, Germany). An overcorrection of the mechanical leg axis crossing the centre of the tibial plateau laterally (55–65% from medial to lateral, depending on the primary diagnosis) was sought and the required correction (in mm) was calculated consecutively. In case, the mechanical lateral distal femur angle (MLDFA) fell below 85° in planning, a concomitant HTO was indicated to avoid excessive postoperative joint line obliquity.

Following lateral distal femoral skin incision and longitudinal split of the iliotibial band, the femoral metaphysis was carefully exposed, bluntly dissecting the vastus lateralis muscle from the intermuscular septum. The biplanar osteotomy planes were marked and a bicortical frontal osteotomy was performed. Next, four axial K-wires, marking the osteotomy wedge to be excised proximally and distally, were placed for axial osteotomy. Consecutively, lateral osteotomy preserving the medial cortex was performed with the hinge located at a 0.5–1 cm distance from the medial cortex. The osteotomy gap was carefully closed applying moderate valgus stress and axial compression. Following temporary fixation, mechanical correction was controlled via fluoroscopy as previously described [12]. A locking compression plate—either PEEK-Power™ Plate (Arthrex Inc., Naples, FL, USA) or TomoFix™ Plate (DePuy Synthes, Raynham, MA, USA)—was used to secure the osteotomy. The postoperative rehabilitation program started on the first postoperative day and depended on the primary diagnosis and the concomitant procedures. For osteotomy with or without ligamentous procedures and meniscal surgery, the standard protocol included a limitation to partial weight bearing (20 kg) from the first postoperative day until 6 weeks postoperatively. Minimal weight bearing (5 kg) was indicated after OATS and no weight bearing was allowed after concomitant cartilage transplantation.

### Follow-up

Patients were routinely followed up in ambulatory care at the authors’ institution at 6 weeks, 12 weeks and 1 year postoperatively and standard radiographs were obtained to evaluate consolidation of the osteotomy plane. In suspicion of a malunion or loss of correction, a computed tomography (CT) or hip–knee–ankle radiograph was conducted, respectively.

### Medial cortical hinge fracture

The presence of a hinge fracture was assessed by two observers (M-C.R and M.J.F.) on each postoperative AP knee radiograph independently. A hinge fracture was defined as a disruption of the medial cortical bone. In cases of disagreement, a third observer (P.W.W.) was consulted to achieve consensus. Furthermore, the fracture morphology of all medial cortical hinge fractures was evaluated. A fracture in line with the osteotomy was classified as *extension* type fracture, while fracture lines diverting proximally or distally of osteotomy orientation were classified as proximal or distal type fractures, respectively.

### Postoperative measurements and hinge position

Standard hip–knee–ankle radiographs acquired preoperatively and standard AP knee radiographs acquired on the first or second postoperative day were used for the analysis. Annotation was conducted by the main observer (M-C.R.) using the picture archiving and communication system (PACS). To assess inter- and intrarater reliability, measurements were performed two times at an interval of 1 month by the main observer (M-C.R.) and additionally by a second observer (P.W.W.) for 20 randomly selected patients.

**Fig. 1:**
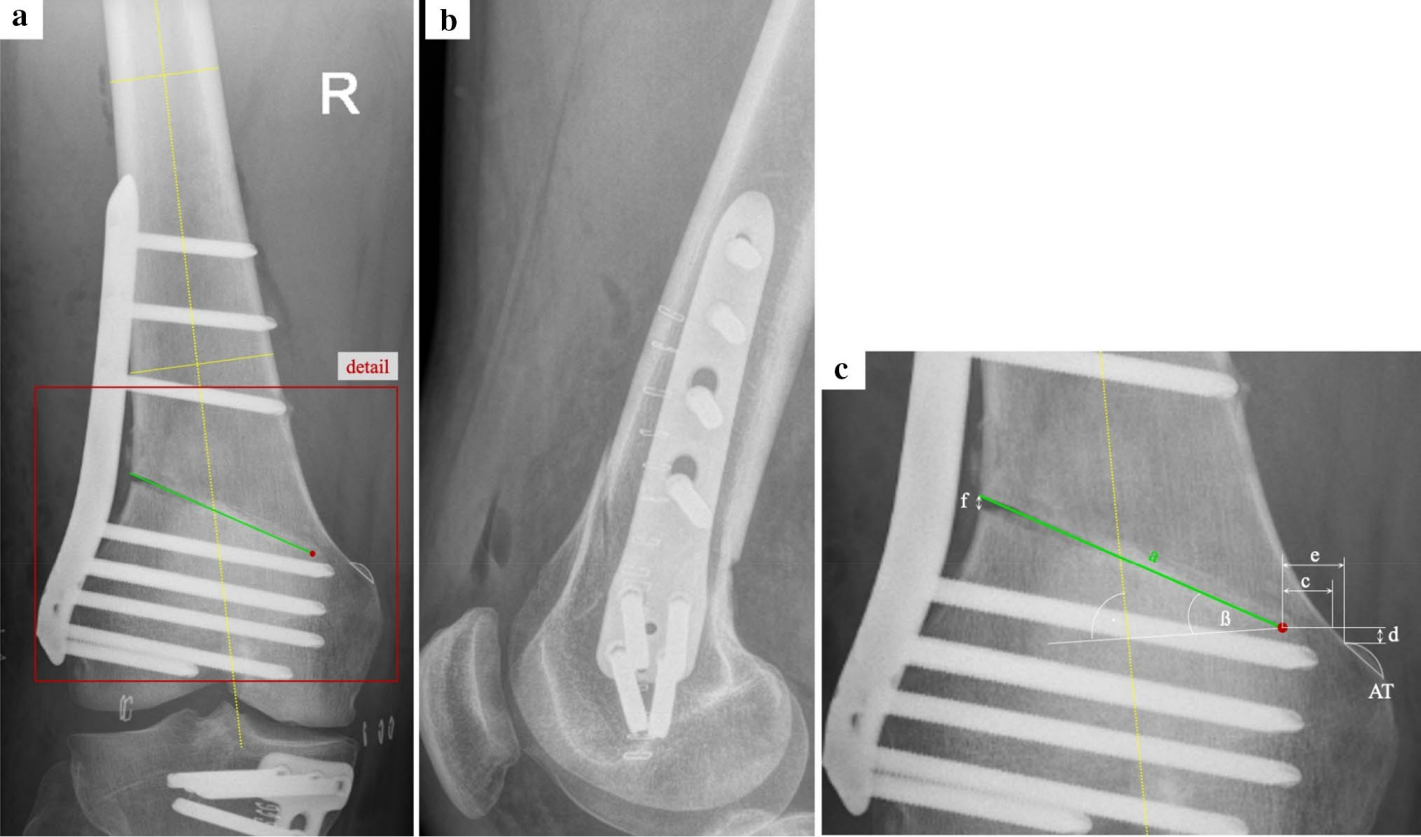
Measurement parameters. **a** Standard anterior–posterior and **b** lateral radiograph of a right knee after lateral closing wedge distal femoral osteotomy using a TomoFix™ (DePuy Synthes, Raynham, MA, USA) locking compression plate. **c** Detailed view of the closing wedge osteotomy cut with the respective measurements. AT, adductor tubercle (grey line); red dot, osteotomy hinge; yellow solid lines, mediolateral diameter femoral cortical bone; yellow dotted line, anatomical axis of the femoral diaphysis, running through the centre of the two yellow solid lines; green line (interval “a”), length of the osteotomy cut; interval “c”, horizontal distance between the medial cortical bone and the osteotomy hinge; interval “d”, vertical distance between the osteotomy hinge and the proximal border of the AT; interval “e”, horizontal distance between the osteotomy hinge and the proximal lateral margin of the AT; interval “f”, distance between the cortices proximal and distal of the osteotomy cut; angle “ß”, inclination of the osteotomy (angle between the proximal osteotomy plane and a line perpendicular to the anatomical axis of the femur)

Measurements were performed as previously described [53] and modified for the CW-technique: on the preoperative hip–knee–ankle radiographs, the preoperative osteotomy planning conducted via mediCAD^®^ software (mediCAD Hectec GmbH, Altdorf, Germany) was identified and the wedge angle (angle α) and the height of the osteotomy wedge (interval “b”) were measured. In the postoperative standard AP radiographs, the length (interval “a”) and the inclination of the osteotomy (angle “ß”) were measured after identification of the osteotomy hinge and the anatomical axis of the femoral diaphysis. To calculate the effective, absolute correction (in mm), a persistent lateral cortical gap (interval “f”) was—if detected—measured and deducted from the preplanned correction. The location of the osteotomy hinge was quantified by measuring the horizontal distance between the medial cortical bone and the hinge (interval “c”) as well as the vertical (interval “d”) and horizontal (interval “e”) distances between the proximal margin of the adductor tubercle (AT) and the hinge point, the position was quantified. To quantify dislocation in case of a hinge fracture, the horizontal distance between the proximal and distal medial cortex was measured. The threshold for a significant dislocation was set at ≥ 2 mm, as previously described for HTO [30]. A comprehensive methodology of the measurements is presented in Fig. 1.

**Fig. 2:**
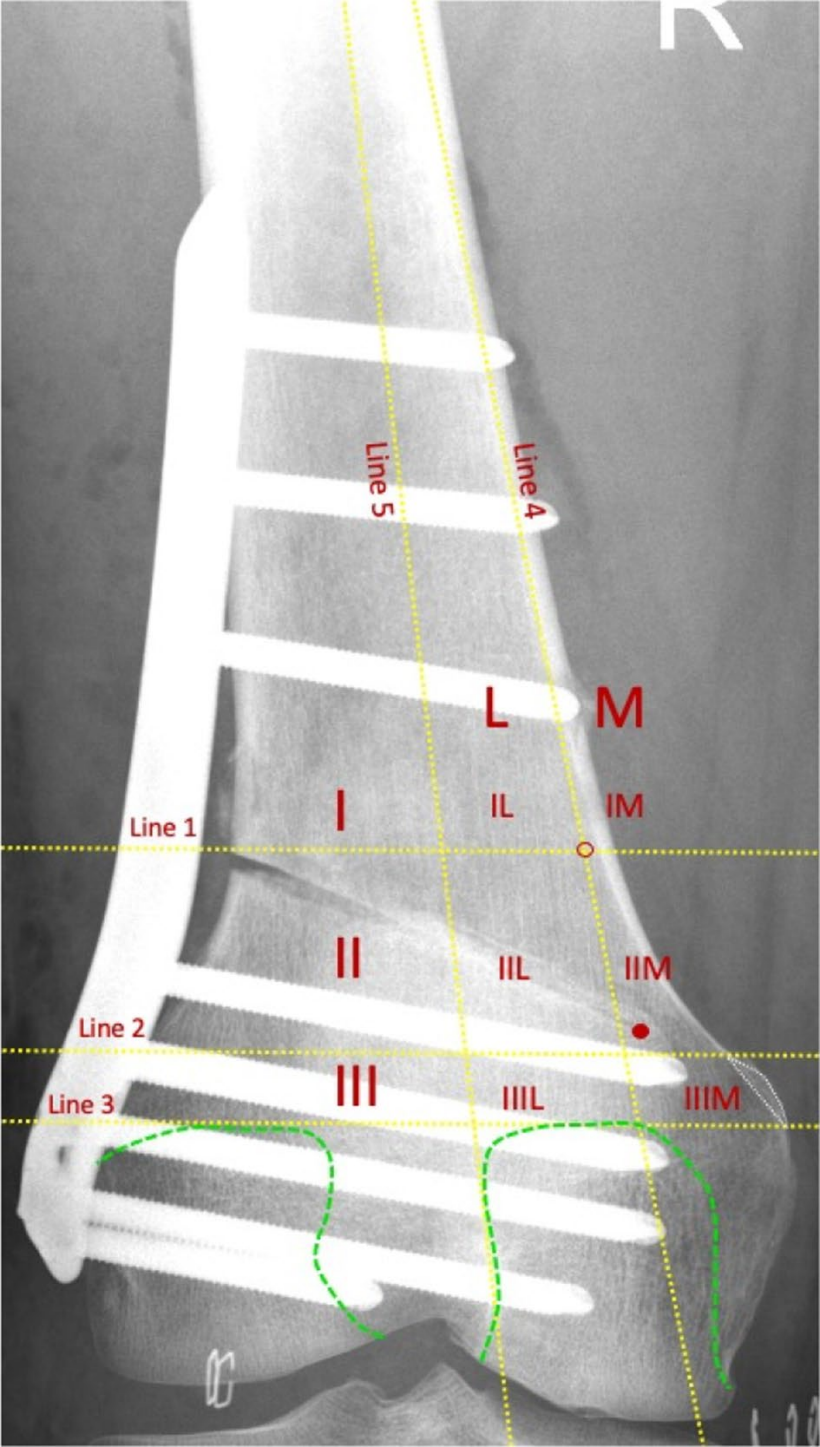
Sector grid. *AT* adductor tubercle (white dotted line), *I* row 1, *II* row 2, *III* row 3, *M* medial column, *L* lateral column, *Red dot* osteotomy hinge, *Green dashed lines* posterior part of the medial and lateral femoral condyle, *Red circle* inflection point, defined as the point at which the distance between the medial cortical bone and Line 4 reaches 2 mm; Line 5, tangential to the lateral facet of the medial femoral condyle; Line 4, tangential to the medial femoral cortical bone; Line 3, tangential to the apices of the posterior part of the medial and lateral femoral condyle; Line 2, parallel to Line 3 and crossing the proximal border of the AT; Line 1, parallel to Line 3 and crossing the inflection point

For consecutive analysis, the two-dimensional position of the osteotomy hinge was assigned to a corresponding sector in a sector grid as previously described for LOW-DFO [53]. Bony landmarks (AT, medial femoral cortical bone, femoral condyles) were used to define two columns (M, L) and three rows (I, II, III) of the grid. The sectors (IL, IIL, IIIL, IIM, and IIIM) were defined according to their location in the respective rows and columns. A detailed description of the five-sector grid is presented in Fig. 2. The allocation of the hinge position to the corresponding sector was performed by two observers (M-C.R. and P.W.W.) in agreement with each other.

The intraclass correlation values of quantitative measurements showed excellent intrarater (distance “a”, 0,963; distance “c”, 0,968; distance “d”, 0,932; distance “e”, 0,943; angle “ß”, 0,977) and good to excellent interrater reliability (distance „a “, 0,932; distance “c”, 0,919; distance “d”, 0,941; distance “e”, 0,914; angle “ß”, 0,857).

### Risk factor analysis

Constitutional and technical risk factors predisposing for the incidence of consecutive complications after medial cortical fracture in LCW-DFO were analysed. The size of our study population statistically limited the number of risk factors to be evaluated, since repeatedly testing an excessive number of factors on a single dataset predisposes for the occurrence of Type 1 (false-positive) errors. Therefore, we selected the following preoperative factors a priori for assessment of our secondary hypothesis in this study: constitutional factors (BMI, age, sex, smoker), fracture morphology, quantitative hinge position measurements, concomitant procedures, postoperative dislocation, and implant type.

### Statistical analysis

A total sample size of 76 subjects to detect a difference of 1.5 mm of a primary endpoint measurement, the absolute correction, at a calculated effect size of 0.66 to achieve a statistical power of 0.8 was determined in an a priori power analysis, performed with G*Power (Erdfelder, Faul, Buchner, Lang, HHU Düsseldorf, Düsseldorf, Germany) [10].

Statistical analysis was performed using SPSS software version 26.0 (IBM-SPSS, New York, USA). Continuous variables were reported as mean ± standard deviation. The distribution of continuous variables in the study collective was categorised via Shapiro–Wilk Test. Categorical variables were reported as count and percentages. According to their respective distribution, continuous variables were compared employing a parametric unpaired *t* test or the non-parametric Mann–Whitney *U* test. Categorical variables were compared performing the binary Fisher’s exact test or the Chi-square test, as statistically appropriate. The level of significance was set at *p* < 0.05.

To determine the risk of creating a medial cortical hinge fracture, a multivariable logistic regression was performed. The dependent variable was defined as the incidence of “medial cortical hinge fracture” (yes vs. no). Quantitative variables describing the location of the hinge position, that demonstrated a significant difference (*p* < 0.05) between the two groups (hinge fracture vs. no hinge fracture) in univariate analysis, were defined as independent variable and were used as the covariates.

## Results

### Incidence and fracture morphology

**Fig. 3:**
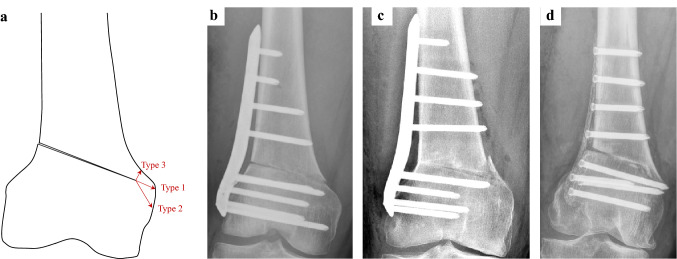
Morphology and classification of medial cortical hinge fractures. **a** Schematic illustration of the three different fracture types. **b** Type 1 fracture, extension of the osteotomy plane. **c** Type 2 fracture, distal to the osteotomy hinge. **d** Type 3 fracture, proximal to the osteotomy hinge

A medial cortical hinge fracture was detected in 38 (48%) of the cases. Assessing the fracture morphology, 26 (68%) of the fractures were classified as extension type fractures, while 8 (21%) were categorised as proximal and 4 (11%) as distal fractures (Fig. 3).

### Risk factors for sustaining a hinge fracture

The association of technical and anatomical factors characterising the osteotomy cut and hinge position with the incidence of a hinge fracture is presented in Table 2.

**Table 2:** Categorical variables are presented as count and percentage; continuous variables are presented as mean ± standard deviation; positive values of distance “d” indicated a hinge position proximal to the adductor tubercle (AT), while negative values indicated a hinge position distal to the proximal margin of the AT

Variable		Hinge fracture			*p* value
			No	Yes	
Number of included patients, *n*			41	38	–
Age^a^ (years)			45 ± 13	49 ± 12	n.s
BMI (kg/m^2^)			28 ± 5	29 ± 4	n.s
Sex					n.s
Female			17 (42%)	10 (26%)	
Male			24 (58%)	28 (74%)	
Laterality					n.s
Left			24 (59%)	22 (58%)	
Right			17 (41%)	16 (42%)	
Distance a [mm]			53.1 ± 10.9	57.7 ± 9.6	0,049*
Distance b [mm]			6.5 ± 1.9	7.9 ± 3	0,040*
Distance c [mm]			10,4 ± 5,2	9.0 ± 6.3	n.s
Distance d [mm]			7.7 ± 6.7	6.8 ± 9.6	n.s
Distance e [mm]			13.8 ± 6.6	11.6 ± 8.4	n.s
Distance f [mm]			1.48 ± 1.22	1.2 ± 0.9	n.s
Absolute correction [mm]			5 ± 2	7 ± 3	0,015*
Α [°]			7 ± 2.1	7,8 ± 3.1	n.s
ß [°]			26.6 ± 6,5	25 ± 6.3	n.s
Sector grid vertical orientation					n.s
I			4 (10%)	4 (11%)	
II			30 (74%)	25 (66%)	
III			7 (17%)	9 (24%)	
Sector grid horizontal orientation					0,049*
M			8 (19%)	16 (42%)	
L			33 (81%)	22 (58%)	

*BMI* body-mass-index, *HTO-MOW* high tibial osteotomy medial open wedge, *OATS* osteochondral autograft transfer system, *n.s.* non-significant;

^a^Age at surgery

^b^Total number of patients exceeds 79 (total study group), as certain patients underwent more than one concomitant procedure

^*^ Statistically significant difference between groups (level of significance, *p* < 0.05)

For the osteotomy cut, an increased length of the osteotomy in mm (53.1 ± 10.9 vs. 57.7 ± 9.6; *p* = 0.049), an increased preplanned correction in mm (6.5 ± 1.9 vs. 7.9 ± 3; *p* = 0.040) as well as an increased absolute postoperative correction in mm (5 ± 2 vs. 7 ± 3; *p* = 0.015) were shown to be associated with the incidence of a medial cortical hinge fracture. Neither the angle of the excised wedge (α), nor the inclination of the osteotomy cut (ß), nor the distance of the hinge to the medial cortex (c) nor the craniocaudal position of the hinge (in relation to the AT) (d) nor the mediolateral distance of hinge to the AT (e) were shown to significantly influence the incidence of a medial cortical hinge fracture in our collective (*p* = n.s., respectively; for details, Table 2). The incidence of a medial cortical hinge fracture was significantly influenced by the horizontal location of the hinge in the sector grid—a higher incidence in column M compared to column L (*p* = 0.049) was detected—while neither the vertical location nor a specific two-dimensional sector were shown to exhibit a significant influence (*p* = n.s., respectively; for details, Table 2). A multivariable logistic regression model for the incidence of a “medial cortical hinge fracture” (model: *p* = 0.003) showed statistical significance for the absolute correction (*p* = 0.014). An increase of absolute correction by 1 mm increased the risk of creating a medial cortical hinge fracture by 32%.

### Complications

Complications occurred in 7 (9%) of the cases. The incidence of malunions during follow-up was 6 (8%) across the entire patient collective. While not statistically significant (*p* = 0.10), these malunions tended to occur more frequently in the cases with a concomitant medial cortical hinge fracture (*n* = 5; 13%) compared to cases with an intact medial cortical bone (*n* = 1; 2%) (2). A detailed description of the associated complications in the study collective can be found in Table 3.

**Table 3:** Categorical variables are presented as count and percentage; continuous variables are presented as mean ± standard deviation

Complication	Hinge fracture		*p* value
	No	Yes	
None, *n* (%)	40 (98%)	32 (84%)	n.s
Bleeding, *n* (%)	0 (0%)	1 (3%)	n.s
Infection, *n* (%)	0 (0%)	0 (0%)	n.s
Delayed union, *n* (%)	0 (0%)	1 (3%)	n.s
Non-union, *n* (%)	1 (2%)	4 (11%)	n.s
Screw loosening, *n* (%)	0 (0%)	2 (5%)	n.s
Loss of correction, *n* (%)	0 (0%)	2 (5%)	n.s

The total number of complications exceeds number of cases with complications, as two patients suffered more than one complication

**Fig. 4:**
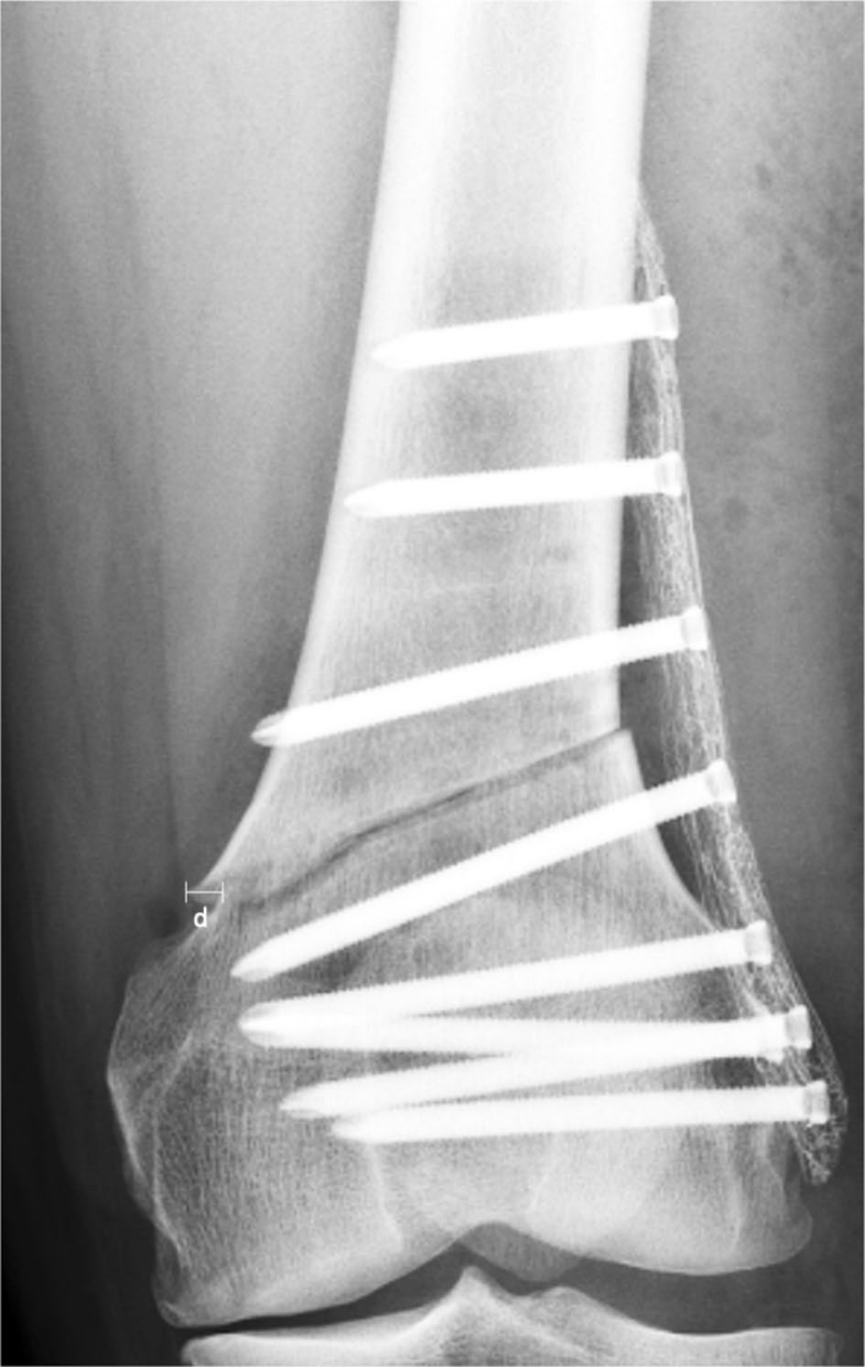
Mediolateral dislocation after medial cortical hinge fracture. A standard AP radiograph taken on the first postoperative day after biplane LCW-DFO using a TomoFix™ (DePuy Synthes, Raynham, MA, USA) locking compression plate is showing a mediolateral dislocation of > 2 mm in the coronal plane caused by a medial cortical hinge fracture. Distance d; horizontal distance between the proximal and distal medial cortex

Furthermore, clinical, technical and anatomical risk factors for malunion after LCW-DFO were analysed in the subgroup of patients with a hinge fracture. It was shown that a mediolateral dislocation of the medial cortex of > 2 mm (Fig. 4) in the coronal plane on the postoperative standard AP radiograph predisposed for the incidence of malunion (*p* = 0.048; relative risk: 1.33; odds ratio: 13.31). A mediolateral dislocation of > 2 mm in the coronal plane could be detected in 100% of the cases with malunion. In our collective, demographic factors, smoker status, hinge location, fracture morphology and implant type did not significantly influence the rate of complications affecting osteotomy consolidation. A detailed risk factor analysis can be found in Table 4.

**Table 4:** Risk factor analysis for malunion in the subgroup of cases with a hinge fracture

Variable	Malunion		*p* value
	No	Yes	
Number of patients, *n* (%)	33 (87%)	5 (13%)	–
Age^a^ (years)	50 ± 12	48 ± 7	n.s
BMI (kg/m^2^)	28.9 ± 4.6	29.2 ± 3.1	n.s
Smoker			n.s
No	22 (76%)	4 (80%)	
Yes	7 (24%)	1 (20%)	
n.a. (*n* = 4)			
Sex			n.s
Female	9 (27%)	1 (20%)	
Male	24 (73%)	4 (80%)	
Sector grid vertical orientation			n.s
I	3 (9%)	1 (20%)	
II	22 (67%)	3 (60%)	
III	8 (24%)	1 (20%)	
Sector grid horizontal orientation			n.s
M	14 (42%)	2 (40%)	
L	19 (58%)	3 (60%)	
Fracture morphology			n.s
Type 3 (proximal)	7 (21%)	1 (20%)	
Type 1 (extension)	22 (67%)	4 (90%)	
Type 2 (distal)	4 (12%)	0 (0%)	
Dislocation > 2 mm			0,048*
No	18 (55%)	0 (0%)	
Yes	15 (45%)	5 (100%)	
Implant type			n.s
Tomofix™	24 (73%)	3 (60%)	
PEEK-Power™	9 (27%)	2 (40%)	

Continuous variables are presented as mean ± standard deviation (range); categorical variables are presented as count and percentage

*BMI* body-mass-index, *n.s.* non-significant, *n.a.* not available

^a^Age at surgery

## Discussion

The present study constitutes two main findings. First, the presence of a hinge fracture after LCW-DFO is common (48%) and three morphological types can be differentiated in the largest collective of LCW-DFOs in the literature to date. Furthermore, the secondary hypothesis could be confirmed, as the extent of surgical alignment correction and a hinge position close to the medial cortex predispose for medial cortical hinge fracture and a mediolateral dislocation of the medial cortex > 2 mm is significantly associated with malunion.

In the literature, lateral cortical hinge fractures have been identified as a common complication following MOW-HTO, occurring in 18–50% [7, 16, 22–24, 28, 30, 33, 34, 40, 49] and substantial evidence has been published identifying hinge fractures as an independent risk factors for inadequate bone healing [7, 16, 30]. While less extensively investigated to date, similar data have been published for hinge fractures following DFO, with an incidence of 39–46% reported for the LOW- [53], 48% for the MCW- [14] and 31% for the LCW-technique [36] in accordance with the incidence reported in the present study.

In MOW-HTO, a fracture classification established by Takeuchi et al. [49], that differentiates three morphological types (extension/proximal/distal), is well accepted [16, 33, 49]. Similarly, a classification has been published for LOW-DFO [53]. A study published by Nakayama et al. in LCW-DFO reports only two distinct fracture types (proximal / extension) in a study of 11 cases with a hinge fracture. In contrast, in the present study, three distinct fracture types (extension/proximal, distal) could be identified in accordance with the classifications established for HTO and LOW-DFO [49, 53], which may possibly be explained by a minimization of the risk for a type II error by a threefold greater power in the present study. In accordance with data reported after HTO [16, 23, 40, 49], LOW- [53] and LCW-DFO [36], an extension fracture was the most frequently identified morphological type.

As an accepted risk factor for malunion after MOW-HTO [7, 16, 30], substantial efforts have been made to mitigate the risk of unstable cortical hinge fracture in HTO [17, 33, 40], MCW-DFO [21] and LOW-DFO [53] by avoiding technical and constitutional risk factors.

In the present study, an increased height of the excised wedge as well as a hinge location close to the medial cortex have been identified as risk factors associated with a medial cortical hinge fracture. This is in accordance with data from HTO, that associated larger osteotomy gaps and location close to the opposite cortex with an increased risk for fracture [20, 24, 32, 33, 38, 47].

Interestingly, the wedge angle (A) was not found to significantly affect the risk for sustaining a hinge fracture, though trigonometrically related to wedge height (b). This seeming controversy may potentially be explained by the wedge angles inversely proportional relation the osteotomy length (a) and thus mediolateral position of the hinge—that, respectively, increase the risk for hinge fracture (*p* = 0.049)—and thus confounds the distribution of the wedge angles between the hinge fracture groups (“no” vs. “yes”). While the accuracy of the placement of the osteotomy cut in the sub-millimetre range is potentially unrealistic intraoperatively [50], the technical factors identified to reduce the risk of a hinge fracture may serve as an orientational guideline in planning the osteotomy (consideration of a double level approach in cases of excessive wedge height and avoidance of an overly lateral placement of the hinge) rather than absolute thresholds based on this retrospective study.

While a lower incidence of hinge fractures at the level or distal of the proximal margin of the AT was reported for LOW-DFO [53], no statistical significance for this finding could be constituted in the present study—possibly due the incidence of a type II error and a heterogeneity in the surgical technique due to the long inclusion period. The biomechanical properties of the lateral femoral condyle in response to an LCW osteotomy yet remain to be investigated.

While extensive investigations in HTO associated certain fracture configurations such as Takeuchi type II and III fractures with an instability of the osteotomy site [16, 33, 49], there is a paucity of evidence for DFO [39]. In accordance with the findings of the present study, studies investigating lateral DFO do not report the specific fracture morphology to be associated with an increased risk for instability [36, 53]. However, this study is the first to report an increased incidence of malunion in association with a mediolateral dislocation > 2 mm on the postoperative AP radiograph after lateral DFO. Accordingly, displaced cortical hinge fractures in HTO have been associated with an increased incidence of adverse events [28]. Hence, similar to HTO, a mediolateral dislocation may correspond to a clinically relevant instability at the osteotomy site [16, 31–33, 49]. For clarification, further studies in a larger collective correlating fracture morphology and cortical fracture location to a mediolateral dislocation are warranted. A loss of correction was shown in 5.3% in patients with a medial cortical fracture, a complication known after hinge fractures in high tibial osteotomy with an incidence between 1 and 15% [16, 28, 32, 33] and varising DFO of the valgus knee with an heterogenous incidence of 3–23% [8, 14, 25, 54].

We, therefore, recommend that a hinge fracture in LCW-DFO—if noticed intraoperatively—should be encountered by additional medial plate fixation, while—if recognised postoperatively—the weight bearing regime should be delayed to avoid complications [36], as commonly practiced in HTO [16, 23, 28, 38].

### Limitations

While this study does demonstrate interesting findings, it is not without limitations. First, the incidence and configuration of medial cortical hinge fractures were assessed on standard postoperative AP radiographs, while previous studies postulated an even higher detection of this complication via CT [23]. As postoperative CT scans were not performed at the senior authors’ institution due to the increased radiation dose the patient is exposed to, we are aware that the incidence of medial cortical hinge fractures may be underreported. Second, radiological evaluation was conducted in the first 2 days postoperatively. Hence, the incidence of cortical disruptions beyond this time point could not be assessed. Third, as incidence of and risk factors for hinge fractures and complications were elected as primary and secondary endpoints, no clinical scores were included. Thus, the impact of this complication on the clinical outcome is not reported, as this exceeded the scope of the study. Fourth, certain *p* values fall just below the threshold of statistical significance, thus limiting the strength of the conclusion. Fifth, as the study inherits the associated biases of a retrospective design, further biomechanical and prospective clinical evidence may improve the understanding of technical risk factors for sustaining a hinge fracture after LCW-DFO.

## Conclusion

In the largest collective of LCW-DFO to date, medial cortical hinge fractures are a common finding. An increased risk of sustaining a hinge fracture has to be expected with increasing osteotomy wedge height and a far medial location of the hinge. To minimise the risk of complications, dislocation after medial cortical disruption should be avoided since mediolateral dislocation greater 2 mm was associated with malunion. In these cases, additional medial plate fixation or a more restrictive rehabilitation protocol should be considered.

## Abbreviations

AT: Adductor tubercle
BMI: Body-mass-index
CT: Computed tomography
CW: Closing wedge
DFO: Distal femoral osteotomy
HTO: High tibial osteotomy
LCW: Lateral closing wedge
LOW: Lateral open wedge
MCW: Medial closing wedge
MLDFA: Mechanical lateral distal femur angle
MOW: Medial open wedge
OATS: Osteochondral autograft transplant

## References

[1] Ackermann J, Merkely G, Arango D, Mestriner AB, Gomoll AH (2020). The effect of mechanical leg alignment on cartilage restoration with and without concomitant high tibial osteotomy. Arthroscopy.

[2] Babis GC, An KN, Chao EY, Rand JA, Sim FH (2002). Double level osteotomy of the knee: a method to retain joint-line obliquity. Clinical results. J Bone Jt Surg Am.

[3] Batista BB, Volpon JB, Shimano AC, Kfuri M (2015). Varization open-wedge osteotomy of the distal femur: comparison between locking plate and angle blade plate constructs. Knee Surg Sports Traumatol Arthrosc.

[4] Cameron JI, McCauley JC, Kermanshahi AY, Bugbee WD (2015). Lateral opening-wedge distal femoral osteotomy: pain relief, functional improvement, and survivorship at 5 years. Clin Orthop Relat Res.

[5] Cao Z, Mai X, Wang J, Feng E, Huang Y (2018). Unicompartmental knee arthroplasty vs. high tibial osteotomy for knee osteoarthritis: a systematic review and meta-analysis. J Arthroplasty.

[6] Dewilde TR, Dauw J, Vandenneucker H, Bellemans J (2013). Opening wedge distal femoral varus osteotomy using the Puddu plate and calcium phosphate bone cement. Knee Surg Sports Traumatol Arthrosc.

[7] Dexel J, Fritzsche H, Beyer F, Harman MK, Lützner J (2017). Open-wedge high tibial osteotomy: incidence of lateral cortex fractures and influence of fixation device on osteotomy healing. Knee Surg Sports Traumatol Arthrosc.

[8] Edgerton BC, Mariani EM, Morrey BF (1993). Distal femoral varus osteotomy for painful genu valgum: a five-to-11-year follow-up study. Clin Orthop Relat Res.

[9] Ekeland A, Nerhus TK, Dimmen S, Heir S (2016). Good functional results of distal femoral opening-wedge osteotomy of knees with lateral osteoarthritis. Knee Surg Sports Traumatol Arthrosc.

[10] Faul F, Erdfelder E, Lang AG, Buchner A (2007). G*Power 3: a flexible statistical power analysis program for the social, behavioral, and biomedical sciences. Behav Res Methods.

[11] Felson DT, Niu J, Gross KD, Englund M, Sharma L, Cooke TD (2013). Valgus malalignment is a risk factor for lateral knee osteoarthritis incidence and progression: findings from the Multicenter Osteoarthritis Study and the Osteoarthritis Initiative. Arthritis Rheum.

[12] Feucht MJ, Mehl J, Forkel P, Imhoff AB, Hinterwimmer S (2017). Distal femoral osteotomy using a lateral opening wedge technique. Oper Orthop Traumatol.

[13] Feucht MJ, Winkler PW, Mehl J, Bode G, Forkel P, Imhoff AB (2020). Isolated high tibial osteotomy is appropriate in less than two-thirds of varus knees if excessive overcorrection of the medial proximal tibial angle should be avoided. Knee Surg Sports Traumatol Arthrosc.

[14] Forkel P, Achtnich A, Metzlaff S, Zantop T, Petersen W (2015). Midterm results following medial closed wedge distal femoral osteotomy stabilized with a locking internal fixation device. Knee Surg Sports Traumatol Arthrosc.

[15] Fürmetz J, Patzler S, Wolf F, Degen N, Prall WC, Soo C (2020). Tibial and femoral osteotomies in varus deformities: radiological and clinical outcome. BMC Musculoskelet Disord.

[16] Goshima K, Sawaguchi T, Shigemoto K, Iwai S, Nakanishi A, Inoue D (2019). Large opening gaps, unstable hinge fractures, and osteotomy line below the safe zone cause delayed bone healing after open-wedge high tibial osteotomy. Knee Surg Sports Traumatol Arthrosc.

[17] Han SB, Lee DH, Shetty GM, Chae DJ, Song JG, Nha KW (2013). A "safe zone" in medial open-wedge high tibia osteotomy to prevent lateral cortex fracture. Knee Surg Sports Traumatol Arthrosc.

[18] Harris JD, McNeilan R, Siston RA, Flanigan DC (2013). Survival and clinical outcome of isolated high tibial osteotomy and combined biological knee reconstruction. Knee.

[19] Kahlenberg CA, Nwachukwu BU, Hamid KS, Steinhaus ME, Williams RJ (2017). Analysis of outcomes for high tibial osteotomies performed with cartilage restoration techniques. Arthroscopy.

[20] Kessler OC, Jacob HA, Romero J (2002). Avoidance of medial cortical fracture in high tibial osteotomy: improved technique. Clin Orthop Relat Res.

[21] Kim TW, Lee MC, Cho JH, Kim JS, Lee YS (2019). The ideal location of the lateral hinge in medial closing wedge osteotomy of the distal femur: analysis of soft tissue coverage and bone density. Am J Sports Med.

[22] Kim TW, Lee SH, Lee JY, Lee YS (2019). Effect of fibular height and lateral tibial condylar geometry on lateral cortical hinge fracture in open wedge high tibial osteotomy. Arthroscopy.

[23] Lee OS, Lee YS (2018). Diagnostic value of computed tomography and risk factors for lateral hinge fracture in the open wedge high tibial osteotomy. Arthroscopy.

[24] Lee SS, Celik H, Lee DH (2018). Predictive factors for and detection of lateral hinge fractures following open wedge high tibial osteotomy: plain radiography versus computed tomography. Arthroscopy.

[25] Liska F, Haller B, Voss A, Mehl J, Imhoff FB, Willinger L (2018). Smoking and obesity influence the risk of nonunion in lateral opening wedge, closing wedge and torsional distal femoral osteotomies. Knee Surg Sports Traumatol Arthrosc.

[26] Liu JN, Agarwalla A, Garcia GH, Christian DR, Gowd AK, Yanke AB (2019). Return to sport and work after high tibial osteotomy with concomitant medial meniscal allograft transplant. Arthroscopy.

[27] Liu JN, Agarwalla A, Gomoll AH (2019). High tibial osteotomy and medial meniscus transplant. Clin Sports Med.

[28] Martin R, Birmingham TB, Willits K, Litchfield R, Lebel ME, Giffin JR (2014). Adverse event rates and classifications in medial opening wedge high tibial osteotomy. Am J Sports Med.

[29] Matsumoto T, Hashimura M, Takayama K, Ishida K, Kawakami Y, Matsuzaki T (2015). A radiographic analysis of alignment of the lower extremities–initiation and progression of varus-type knee osteoarthritis. Osteoarthritis Cartilage.

[30] Meidinger G, Imhoff AB, Paul J, Kirchhoff C, Sauerschnig M, Hinterwimmer S (2011). May smokers and overweight patients be treated with a medial open-wedge HTO? Risk factors for non-union. Knee Surg Sports Traumatol Arthrosc.

[31] Miller BS, Dorsey WO, Bryant CR, Austin JC (2005). The effect of lateral cortex disruption and repair on the stability of the medial opening wedge high tibial osteotomy. Am J Sports Med.

[32] Miller BS, Downie B, McDonough EB, Wojtys EM (2009). Complications after medial opening wedge high tibial osteotomy. Arthroscopy.

[33] Nakamura R, Komatsu N, Fujita K, Kuroda K, Takahashi M, Omi R (2017). Appropriate hinge position for prevention of unstable lateral hinge fracture in open wedge high tibial osteotomy. Bone Jt J.

[34] Nakamura R, Komatsu N, Murao T, Okamoto Y, Nakamura S, Fujita K (2015). The validity of the classification for lateral hinge fractures in open wedge high tibial osteotomy. Bone Jt J.

[35] Nakayama H, Iseki T, Kanto R, Kambara S, Kanto M, Yoshiya S (2020). Physiologic knee joint alignment and orientation can be restored by the minimally invasive double level osteotomy for osteoarthritic knees with severe varus deformity. Knee Surg Sports Traumatol Arthrosc.

[36] Nakayama H, Kanto R, Onishi S, Kambara S, Amai K, Yoshiya S (2020). Hinge fracture in lateral closed-wedge distal femoral osteotomy in knees undergoing double-level osteotomy: assessment of postoperative change in rotational alignment using CT evaluation. Knee Surg Sports Traumatol Arthrosc.

[37] Nakayama H, Schröter S, Yamamoto C, Iseki T, Kanto R, Kurosaka K (2018). Large correction in opening wedge high tibial osteotomy with resultant joint-line obliquity induces excessive shear stress on the articular cartilage. Knee Surg Sports Traumatol Arthrosc.

[38] Nelissen EM, van Langelaan EJ, Nelissen RG (2010). Stability of medial opening wedge high tibial osteotomy: a failure analysis. Int Orthop.

[39] Nha KW, Chang YS, Shon OJ, Shim BJ, Lee JS, Song JS (2019). Where is the target point to prevent cortical hinge fracture in medial closing-wedge distal femoral varus osteotomy?. J Knee Surg.

[40] Ogawa H, Matsumoto K, Akiyama H (2017). The prevention of a lateral hinge fracture as a complication of a medial opening wedge high tibial osteotomy: a case control study. Bone Jt J.

[41] Pietsch M, Hochegger M, Winkler M, Sandriesser S, Freude T, Augat P (2019). Opening-wedge osteotomies of the distal femur: minor advantages for a biplanar compared to a uniplanar technique. Knee Surg Sports Traumatol Arthrosc.

[42] Saragaglia D, Nemer C, Colle PE (2008). Computer-assisted double level osteotomy for severe genu varum. Sports Med Arthrosc Rev.

[43] Saragaglia D, Rouchy RC, Krayan A, Refaie R (2014). Return to sports after valgus osteotomy of the knee joint in patients with medial unicompartmental osteoarthritis. Int Orthop.

[44] Schroter S, Nakayama H, Yoshiya S, Stockle U, Ateschrang A, Gruhn J (2019). Development of the double level osteotomy in severe varus osteoarthritis showed good outcome by preventing oblique joint line. Arch Orthop Trauma Surg.

[45] Sharma L, Song J, Felson DT, Cahue S, Shamiyeh E, Dunlop DD (2001). The role of knee alignment in disease progression and functional decline in knee osteoarthritis. JAMA.

[46] Song JH, Bin SI, Kim JM, Lee BS (2020). What is an acceptable limit of joint-line obliquity after medial open wedge high tibial osteotomy? Analysis based on midterm results. Am J Sports Med.

[47] Spahn G (2004). Complications in high tibial (medial opening wedge) osteotomy. Arch Orthop Trauma Surg.

[48] Strecker W (2007). Planning analysis of knee-adjacent deformities : I. frontal plane deformities. Eur J Trauma Emerg Surg.

[49] Takeuchi R, Ishikawa H, Kumagai K, Yamaguchi Y, Chiba N, Akamatsu Y (2012). Fractures around the lateral cortical hinge after a medial opening-wedge high tibial osteotomy: a new classification of lateral hinge fracture. Arthroscopy.

[50] Tardy N, Steltzlen C, Bouguennec N, Cartier JL, Mertl P, Batailler C (2020). Is patient-specific instrumentation more precise than conventional techniques and navigation in achieving planned correction in high tibial osteotomy?. Orthop Traumatol Surg Res.

[51] van Raaij TM, Takacs I, Reijman M, Verhaar JA (2009). Varus inclination of the proximal tibia or the distal femur does not influence high tibial osteotomy outcome. Knee Surg Sports Traumatol Arthrosc.

[52] Wei J, Gross D, Lane NE, Lu N, Wang M, Zeng C (2019). Risk factor heterogeneity for medial and lateral compartment knee osteoarthritis: analysis of two prospective cohorts. Osteoarthritis Cartilage.

[53] Winkler PW, Rupp MC, Lutz PM, Geyer S, Forkel P, Imhoff AB (2020). A hinge position distal to the adductor tubercle minimizes the risk of hinge fractures in lateral open wedge distal femoral osteotomy. Knee Surg Sports Traumatol Arthrosc.

[54] Wylie JD, Jones DL, Hartley MK, Kapron AL, Krych AJ, Aoki SK (2016). Distal femoral osteotomy for the valgus knee: medial closing wedge versus lateral opening wedge: a systematic review. Arthroscopy.

